# Quantum chemical profiling of protein mutations via fragment-based DFT

**DOI:** 10.3389/fmolb.2026.1770157

**Published:** 2026-02-18

**Authors:** Alejandro Leyva, M. Khalid Khan Niazi

**Affiliations:** 1 Department of Biomedical Engineering, The Ohio State University, Columbus, OH, United States; 2 Department of Pathology, The Ohio State University, Columbus, OH, United States

**Keywords:** AlphaFold, genomics, quantum chemistry, structural biology, whole exome sequencing

## Abstract

Missense mutations have been extensively studied in tumor-suppressing antigens (TP53) to understand oncogenesis within malignant epithelial cells. Using Whole Exome Sequencing (WXS), missense mutations can be profiled into protein sequences to identify the most common variants in tumor samples. Since most mutations arise randomly, it is necessary to isolate those that produce dysfunctional proteins within large cohorts. Using threading and generative algorithms such as AlphaFold and ColabFold, large cohorts of WXS information can be converted into computationally analyzable structures. By evaluating both high- and low-confidence regions in these structures, these antigens can be studied en masse using pipelines that generate analytical inputs for quantum chemistry analysis. We created a pipeline that processed whole-exome sequencing (WXS) data and selected 28 representative TP53 missense mutants from the TCGA-BRCA cohort for quantum-chemical feasibility analysis. These structures were systematically cleaned using tools such as OpenBabel and AmberTools, and each was prepared for Natural Population Analysis (NPA), Electrostatic Potential (ESP) calculations, and Highest and Lowest Occupied Molecular Orbital (HOMO/LUMO) evaluation within Q-Chem. Using this pipeline, population genomics can be integrated with chemoinformatics to analyze electron density concentrations and generate hypothesis-generating electronic descriptors associated with protein dysfunction. By modifying the generated inputs, additional analyses such as Fukui orbitals, chemical shifts, and Raman shifts can also be performed. This provides a computational means to probe electronic properties not readily accessible at scale using experimental techniques.

## Introduction

The TP53 gene is a multi-domain transcription factor responsible for producing antigens that regulate apoptosis, cell cycles, autophagy, metabolism, and DNA repair (Joerger and Alan, 2008; Kastenhuber and Lowe, 2017). Within the 393-amino-acid protein, DNA binding occurs through residues 92–292, producing upregulation or downregulation of specific cascades (Cho et al., 1994).

The tetramerization domain in residues 323–356 is composed of a beta strand (326–333), followed by alpha-helical regions (335–355) that form a tetrameric structure connecting the domains of the p53 protein to form a dimer of dimers (Clore et al., 1994, Miller et al., 1999, Wang et al., 1994; Lalli et al., 2025). The hydrophobic core formed from isoleucine and leucine produces packing interactions around the region, creating the coordinative ability for conformational changes sequential to DNA binding (Clore et al., 1994, Miller et al., 1999, Wang et al., 1994; Lalli et al., 2025). Thr329 and Gly334 form the bridging strand between the beta strand and the alpha-helical region. Arginine and lysine residues at positions 337 and 351 stabilize the salt bridge between asparagine and arginine that corrects the orientation of the alpha helix. The surface of the alpha helices is covered by hydrophobic residues, including phenylalanine, leucine, isoleucine, and alanine. Orientation and parallelism of the helices are coordinated using isoleucine and leucine hydrogen bonds between amines. This produces a combination of van der Waals contacts between leucine and histidine, while forming side-chain interactions between leucine and isoleucine, with glycine residues orienting the angular position of the helix at a 40-degree angle for symmetry. Certain mutations target the salt bridge or hydrophobic packing, preventing transcriptional activation and dimerization of p53 molecules (Bullock and Fersht, 2001; Hulboy and Lozano, 1994).

The first 93 residues of p53 form transactivation domains 1 and 2, and the proline-rich region (62–93) (Hu et al., 2006; Kussie et al., 1996; Kohn and Yves, 2005; Dyson and Wright, 2024; Beaucage and Caruthers, 1981). TAD1 contains residues allowing for phosphorylation, while TAD2 contains short alpha-helical strands that allow transcriptional activation of PUMA, NOXA, and BAX (Hu et al., 2006; Kussie et al., 1996; Kohn and Yves, 2005; Dyson and Wright, 2024; Beaucage and Caruthers, 1981). The proline-rich region is polymorphic, containing PXXP motifs that enable protein binding. Finally, the C-terminal regulatory domain contains large clusters of lysines for ubiquitination, methylation, and acetylation.

Using whole-exome sequencing, extracted DNA from cell lines can be enzymatically fragmented and biotinylated, allowing identification of protein-coding regions (Beaucage and Caruthers, 1981; Bachman, 2013; Hu et al., 2006). Once extracted, these reads are processed in parallel to produce short reads that are compiled by protein region to assemble the amino acid sequence. Once the base-pair content is known, codons can be assembled to produce the full protein.

Typical experimental protocols synthesize mutated DNA by using restriction digestion to excise the protein region of interest (Saiki et al., 1988; Bachman, 2013; Li et al., 2025; Jumper et al., 2021). Using synthetic DNA copies from phosphoramidite chemistry, a heteroduplex forms between mutant and wild-type sequences, and DNA polymerase synthesizes the mutant DNA, which is extracted using methylation-restriction digestion. Computationally, this process is far simpler, as the chemical behavior of proteins can be modeled using the sequence in AlphaFold with developed thresholding algorithms (Evans et al., 2021; Mirdita et al., 2022; Senior et al., 2020; Varadi et al., 2022; Epifanovsky et al., 2021). Using AlphaFold or ColabFold, multiple sequence alignments of protein inputs can be compared against evolutionary variants to predict protein geometry, which is then used to determine bond angles, turns, and secondary structures (Evans et al., 2021; Mirdita et al., 2022; Senior et al., 2020; Varadi et al., 2022; Epifanovsky et al., 2021). These generated protein structure files can be used for molecular dynamics simulations to analyze mechanistic behavior in isolated systems. However, since most mutations are random and functionally invariant, it is difficult to determine which p53 mutants are functionally significant.

Using Q-Chem, electron density distributions can be analyzed to identify electronic perturbations associated with protein mutations (Shao et al., 2006; Helgaker et al., 2000; Szabo and Ostlund, 1996; Parr and Yang, 1989; Achatz and Zambetti, 2016). Q-Chem provides this analysis by solving the Schrödinger equation using density-functional theory and Gaussian basis functions, assuming electrons move within probability densities around nuclei. This can be used to understand transition states and activation states in proteins, and the basis of chemical transitions within these systems. Using Natural Population Analysis, electron densities around nuclei can be quantified and compiled into natural orbitals, yielding stable means to measure charge and electron transfer. Electrostatic potentials and orbital occupations provide a way to track mechanistic differences within proteins for large-scale mutational studies. This provides a means to understand protein misfolding and altered reactivity within the tumor microenvironment. We hypothesize that AlphaFold can be used to generate large cohorts of Protein Data Bank (PDB) structures for quantum-chemical analysis for broad population-genetics studies (Buel and Walters, 2022).

Quantum chemical analysis provides a means to interrogate mutation-induced changes that are not directly observable from sequence or structural prediction alone. While structure-prediction methods capture global folding and domain organization, electronic descriptors derived from Q-Chem quantify how point mutations alter local charge distributions, electrostatic potential, and orbital stability that underlie biochemical interactions. In TP53, these electronic perturbations are relevant to zinc coordination, DNA-binding affinity, and tetramerization, where small changes in charge polarization or electronegativity can disrupt functional interactions without requiring large-scale structural rearrangement. As such, Q-Chem analytics enable a mechanistic interpretation of missense mutations by linking sequence variation to localized electronic effects that contribute to pathogenic dysfunction.

## Materials and methods

Within the TCGA-BRCA cohort, 28 WXS samples were randomly selected to be processed into FASTA files to maximize variability, with one control p53 PDB. The control TP53 sequence was derived from UniProt P04637 (Buel and Kylie, 2022). For the feasibility analysis, the 28 mutants were chosen to demonstrate the capability of the analysis, rather than to demonstrate experimental analysis. Due to the sample size, there is no guarantee of statistical reproducibility or robustness within the analyzed 28 WXS samples. All genetic analyses were performed on the entire BRCA cohort. Each chosen WXS mutant was characterized by its specific substitution and documented. ColabFold was used to generate five biological replicates of each mutant protein structure. Five ColabFold structural predictions were generated for each mutant; a single representative structure was selected for feasibility analysis to limit computational cost. OpenBabel was used to supply adequate hydrogens and ensure neutrality. Q-Chem 6.3.0 and Amber24 were used on the Ohio Supercomputer Center’s Ascend cluster, running on NVIDIA A100 GPUs. AlphaFold 2.3.2 was accessed through ColabFold, in conjunction with Biopython, for structure inference. All base-pair sequences for each mutant in the whole-exome dataset were stored in a CSV named after the file ID, converted into amino-acid labels, and written into FASTA files for AlphaFold generation.

**FIGURE 1:**
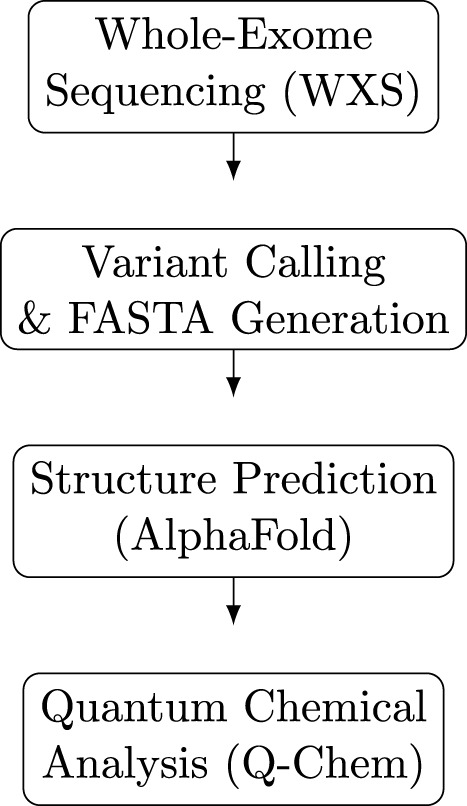
Compact multi-row flowchart summarizing the computational pipeline from WXS data to structural modeling (AlphaFold) and quantum chemical analysis (Q-Chem).

RMSD values and chemical shifts were extracted using BioPDB, and all inputs for sequential Q-Chem analysis were generated using Biopython and BioPDB. Quantum chemical calculations were performed on residue-centered local fragments (±1 residue), ensuring that low-confidence global regions of AlphaFold/ColabFold models do not propagate into the electronic structure analysis. Local fragment-based quantum mechanical calculations do not require explicit propagation of global structure-prediction uncertainty, as the electronic structure is computed deterministically from fixed local geometries and interpreted in a comparative framework. All code is provided on GitHub for accessibility. Each input was then run in Q-Chem using a sequential shell script, in which Natural Population Analyses, Electrostatic Potentials, and orbital occupations (HOMO/LUMO) were computed. Electrostatic potentials were analyzed using the following parameters shown in Table 1, and the workflow for the analysis is shown in Figure 1.

**TABLE 1:** Q-Chem input parameters used for ESP charge calculation, NPA analysis, and HOMO/LUMO orbital extraction.

Parameter	ESP (CHELPG)	NPA	HOMO/LUMO
JOBTYPE	SP	SP	SP
METHOD	B3LYP	B3LYP	B3LYP
BASIS	6–31G*	6–31G*	6–31G*
SCF_CONVERGENCE	7	7	7
MAX_SCF_CYCLES	200	200	200
SCF_ALGORITHM	DIIS_GDM	DIIS_GDM	DIIS_GDM
SYM_IGNORE	1	1	1
NO_REORIENT	1	1	1
CHELPG	TRUE	—	—
NBO	—	1	—
PRINT_ORBITALS	—	—	TRUE

While the 6–31G basis set provides advantageous computational speed and processing for larger sample sizes and analysis, it is acknowledged that the basis set is smaller and reduces accuracy in comparison to conventional basis sets. Specifically, 6–31G lacks diffuse orbitals and angular momentum functions used to describe certain anionic or long-range electrostatic phenomena. In this study, the 6–31G basis set was used for scaling and feasibility testing of Q-Chem analysis on the derived PDB files, rather than to improve the accuracy of the proposed method.

## Results

**FIGURE 2:**
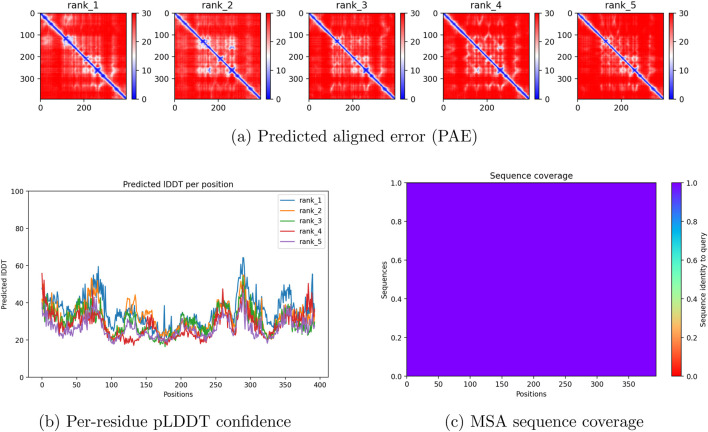
AlphaFold/ColabFold structural prediction outputs for TP53 C176 variants. The full-width panel shows the predicted aligned error (PAE). The bottom row shows per-residue pLDDT confidence scores and the MSA sequence coverage. **(a)** Predicted aligned error (PAE). **(b)** Per-residue pLDDT confidence. **(c)** MSA sequence coverage.

The results of the sequence generation analysis and ColabFold generation show low confidence within the proline-rich regions and a substantial portion of the DNA-binding domain, as shown in Figure 2, likely due to the absence of evolutionary variants. Table 2 displays summary statistics for the cohort, demonstrating a high proportion of missense mutations in addition to most of the mutations being located within the DNA-Binding Domain. This is reasonable, as most cancer-associated mutations are not natural or evolutionarily promoted (Giacomelli et al., 2018). Previous work has demonstrated that AlphaFold2 produces missense point-mutational protein structures that are very similar to the original protein structure (Haley, 2025). In addition, it has been observed that there is no correlation between pLDDT scores and the conservation of proteins such as green fluorescent protein, resulting in similar structures regardless of point mutations (Haley, 2025). In this study, we observe drastically lower pLDDT scores across all selected mutants, reflecting the use of a wider range of missense mutations. Previous studies validating AlphaFold’s reconstruction performance have suggested that only 33% of human proteins in AlphaFold2 have confidence scores lower than 70% (Hosseini et al., 2025). This suggests that the model could not identify an adequate PDB template to model the protein, which resulted in a loss of structure relative to the original wild-type PDB. This led to disconfiguration of several domains, particularly the disruption of the beta-sandwich fold in the DNA-binding domain. This does not suggest contradictory results, as AlphaFold has not been shown to be accurate in reconstructing mutated proteins. Rather, the results demonstrate that the generated mutations are capable of inducing structural changes consistent with those expected from point mutations. This indicates that AlphaFold can model global folding patterns, while quantum chemical analysis is justified to ensure accurate predictions at the electronic level. Low-confidence generated structures are therefore used comparatively to assess local trends in electronic dispersion, without reliance on or interpretation of global structural effects. In addition, there are more than 100 distinct mutations within the cohort that arise in patients with breast cancer, and these sequences originate from malignant cells. Since most mutations were synthetic, the MSA could not identify an evolutionary match to the protein other than the original TP53 sequence. Nevertheless, protein generation was successful.

**TABLE 2:** Summary of TP53 mutation statistics.

Metric	Value
Total TP53 mutation records	256
Unique samples with TP53 mutation	253
Unique TP53 HGVSp variants	127
Missense mutations	204
Nonsense mutations	52
Mutations in TAD1 (1–40)	1
Mutations in TAD2 (41–61)	4
Mutations in PRR (62–93)	1
Mutations in DBD (94–292)	232
Mutations in OD (323–356)	13
Mutations out of canonical range	5
Non-truncating mutations	204
Truncating mutations	52
Conservative missense mutations	62
Non-conservative missense mutations	142
Mutations with unchanged protein length	256

**FIGURE 3:**
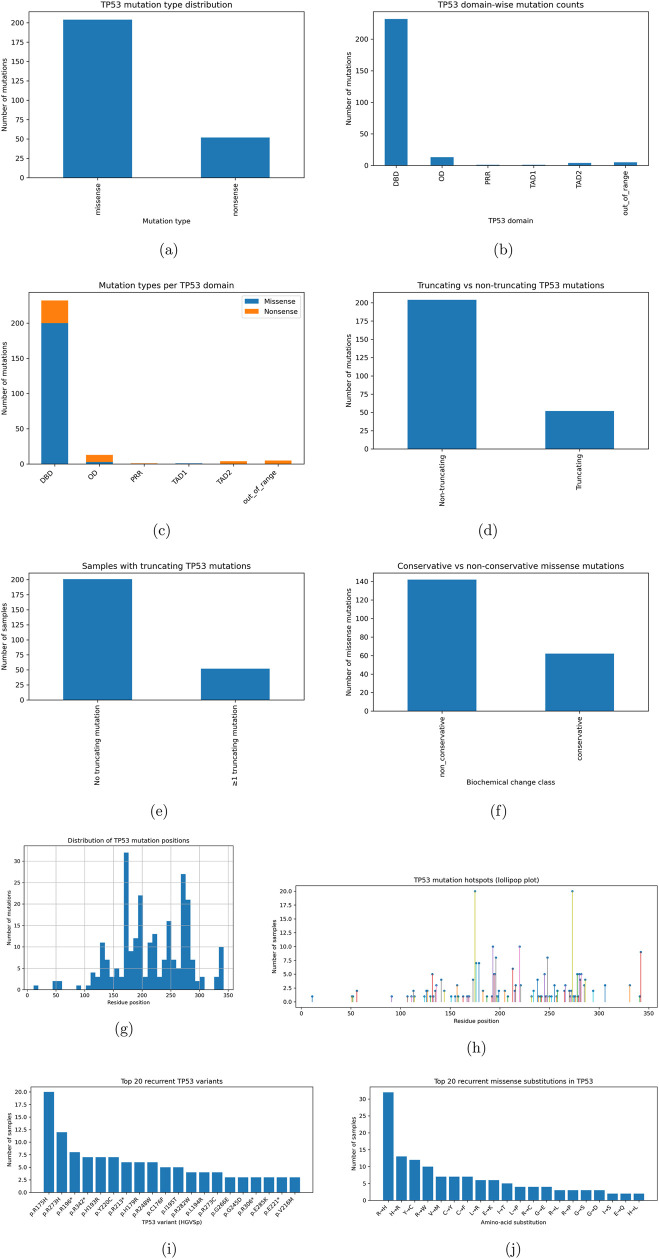
TP53 mutational landscape in the BRCA cohort: global mutation characteristics, truncation status, and biochemical severity. TP53 mutational landscape in the BRCA cohort (ext.): positional distributions, hotspot structure, and recurrent variants. **(a)** Mutation type distribution. **(b)** Domain-wise mutation counts. **(c)** Missense vs. nonsense per domain. **(d)** Truncating vs. non-truncating mutations. **(e)** Samples with truncating TP53 mutations. **(f)** Conservative vs. non-conservative missense. **(g)** Distribution of mutation positions. **(h)** Per-position sample counts (hotspots). **(i)** Top recurrent TP53 variants. **(j)** Top recurrent missense substitutions.

Analytically, most mutations occurred in the DNA-binding domain, as shown in Figure 3, which presents a statistically significant finding that these mutations arise despite the availability of residues. Most mutations in this cohort were missense rather than nonsense mutations, which is statistically more likely based on prior literature on mutational patterns in TP53 (Giacomelli et al., 2018). Most protein mutations were non-truncated, and as expected, all nonsense mutations resulted in shorter proteins.

Biochemically, most of these mutations were evaluated to be non-conservative according to BLOSUM62 scoring and the recorded amino acid substitutions. Most mutations occur within residues 150–200, which correspond to the zinc-binding residues that functionalize TP53 DNA binding (Cho et al., 1994; Joerger and Alan, 2008; Bullock and Fersht, 2001). A secondary hotspot occurs between residues 250–300, corresponding to arginine-binding positions that orient the helical structures of the dimerization complex (Cho et al., 1994; Joerger and Alan, 2008; Bullock and Fersht, 2001). The highest-frequency mutation was R157H, which occurs in the DNA-binding domain and results in downregulation of BMI1 and increased expression of TWIST1, producing epigenetic deformation and unregulated gene expression. Mutant variants such as R273H and R196, which have been found in myeloid acute leukemia and triple-negative breast cancer, respectively, correspond to worse prognosis (Cho et al., 1994; Joerger and Alan, 2008; Bullock and Fersht, 2001; Giacomelli et al., 2018). The most common amino acid substitution is from arginine to histidine, corresponding to the most frequent mutations within the BRCA cohort, all of which destabilize the DNA-binding domain and contribute to proliferation and reduced survival.

**FIGURE 4:**
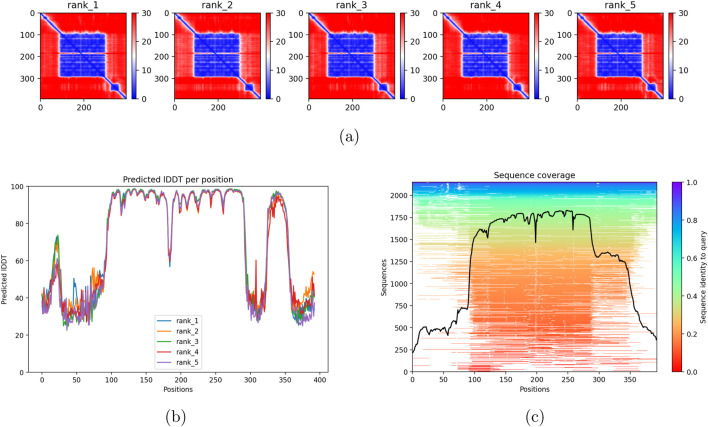
AlphaFold/ColabFold structural prediction outputs for TP53 Wild-Type. The full-width panel shows the predicted aligned error (PAE). The bottom row shows per-residue pLDDT confidence scores and the MSA sequence coverage. **(a)** Predicted aligned error (PAE). **(b)** Per-residue pLDDT confidence. **(c)** MSA sequence coverage.

The results for the wild-type AlphaFold generation in Figure 4 showed high confidence and stable generation of replicates, with evolutionary convergence and high-confidence predictions within the DNA-binding domain. The predicted alignment error demonstrated high confidence across ranks. Wild-type generation of ColabFold structures was successful overall.

**FIGURE 5:**
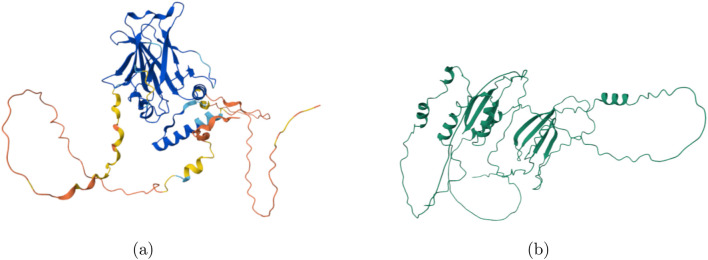
Comparison of the predicted structures for wildtype and mutant TP53. Structural differences are visualized using AlphaFold/ColabFold predicted coordinates. **(a)** Wildtype TP53 structure. **(b)** Mutant TP53 structure.

The display for the wild-type protein shown in Figure 5 was extracted from the Protein Data Bank, and the mutant structures were displayed on the left. Based on the generated structures, the beta-sandwich folds were not conserved, along with a substantial portion of the alpha-helical regions. Low-confidence regions are observed in the unstructured strands, and low-confidence wild-type strands are also shown within proline-rich regions and transactivation domains. The structures were conserved adequately for Q-Chem analysis.

**FIGURE 6:**
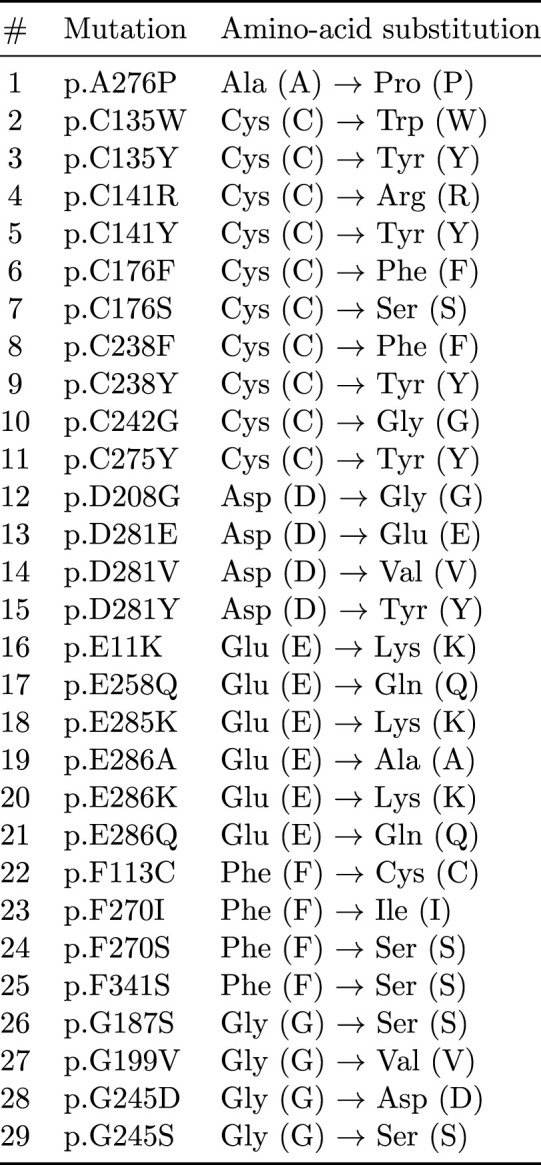
List of TP53 missense mutants analyzed in this study, showing residue position and amino-acid substitution relative to the wild-type sequence.

**FIGURE 7:**
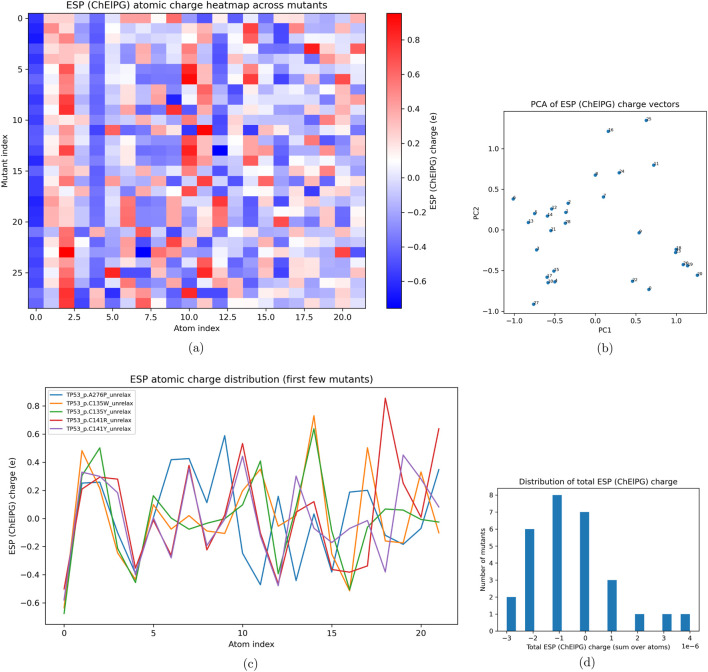
Using sinistrodextral labels, Electrostatic potential (ESP) analyses including **(a)** heatmap, **(b)** PCA, **(c)** first-mutant line plots, and **(d)** histogram of summed ESP charges **(d)**.

Across the 28 mutants shown in Figure 6, a lower electrostatic potential was observed across the cohort, as shown in Figure 7, characterized by substitution of polar amino acids in conserved domains in the DNA-binding region (Cho et al., 1994; Joerger and Alan, 2008; Bullock and Fersht, 2001; Epifanovsky et al., 2021; Shao et al., 2006; Helgaker et al., 2000). High variability was also demonstrated across all mutants, though most mutations resulting in neutral residue substitution had similar variance. Mutants that resulted in substitutions of similar chemical character showed similar variability. Within the mutant cohort were seven cysteine-mutated variants, all within the binding domain that sever the zinc linkage (Cho et al., 1994; Joerger and Alan, 2008; Bullock and Fersht, 2001), one alanine mutant, three aspartate mutations, three glutamate mutations, three phenylalanine mutations, and three glycine mutants. Within this cohort, the glycine mutants typically substituted larger amino acids, while the glutamine mutants resulted in a complete shift from a negatively charged amino acid to a positive one. The phenylalanine mutants showed a shift toward polar thiols, representing a negative shift in charge, with most of these mutations concentrated within the dimerization complex. The cysteine mutations showed a mixed set of potential shifts in both directions, while the aspartate mutations were typically substituted with neutral or hydrophobic amino acids. Across the atomic charge distributions within the fragments of the first five mutants, the cysteine mutants demonstrated an overwhelming similarity at the mutation point and showed correlation with large decreases in ESP (Shao et al., 2006; Helgaker et al., 2000; Szabo and Ostlund, 1996; Parr and Yang, 1989; Achatz and Zambetti, 2016). The distribution across mutants shows a decrease in electric charge, which can be inferred from a subset of mutants, including glycine and cysteine variants, being replaced with polar residues. However, this could also be the result of the natural limitations of ESP analysis, especially at distances below 3 Å and when using a smaller basis set (6–31G) (Hosseini et al., 2025). In this instance, ESP was used as a comparative analysis across all samples to infer structural changes from the ColabFold generation. This suggests that residue polarity shifts the stability and binding capacity of the DNA-binding region within the ColabFold-generated mutants.

**FIGURE 8:**
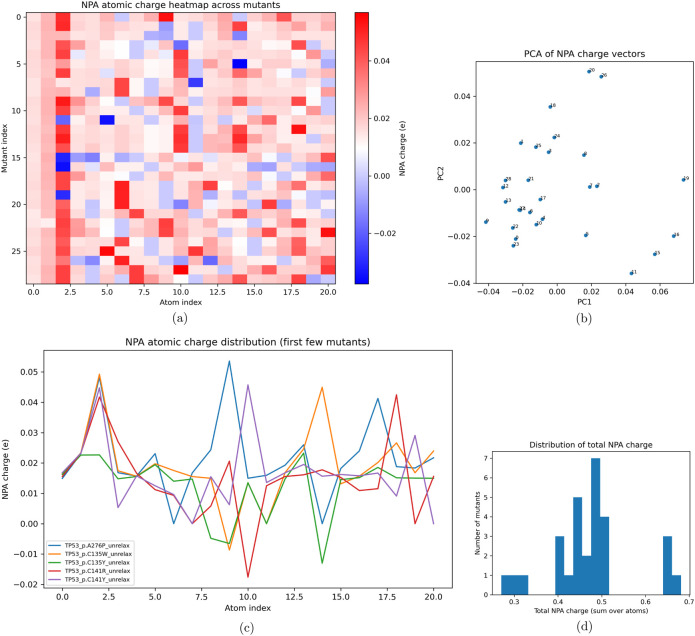
Using sinistrodextral labels, Natural Population Analysis (NPA) charge analyses including **(a)** heatmap, **(b)** PCA, **(c)** first-mutant line plots, and **(d)** histogram of summed NPA charges.

Within the mutation fragments, the electronic distributions in Figure 8 were sparse. Across the first few mutants, the Natural Population Analysis demonstrated that each of the four cysteine mutations showed unique differences in charge across the atoms within the fragment, yet heavy convergence was noted at the third atom index, likely a component shared across all mutants (Shao et al., 2006; Helgaker et al., 2000; Szabo and Ostlund, 1996; Parr and Yang, 1989; Achatz and Zambetti, 2016). The distribution of charge across the NPA output contained outliers, though a high frequency of observations between 0.4 and 0.5, when summed over atoms, demonstrated high polarization within the fragment, with several mutants showing greater polarization approaching 0.7. The electron distributions in these mutants are overwhelmingly undersaturated, resulting in high polarization (Shao et al., 2006; Helgaker et al., 2000; Szabo and Ostlund, 1996; Parr and Yang, 1989; Achatz and Zambetti, 2016). This is consistent with the mechanisms by which the orientation of the DNA-binding domain collapses under disrupted zinc coordination and altered charge density.

**FIGURE 9:**
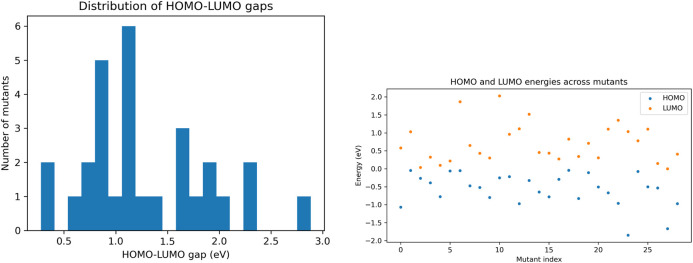
Using sinistrodextral labels, Quantum mechanical electronic structure metrics including **(A)** HOMO–LUMO energy gap histogram and **(B)** HOMO–LUMO scatter distribution across mutants.

Across the HOMO and LUMO distribution in Figure 9, a high frequency of mutants displayed an overwhelmingly electronically stable distribution, which presents a limitation of the analysis. While ESP and NPA results conclude that the electrostatic potential is reduced and that the electron distribution across fragments is sparse, HOMO/LUMO analysis indicated relatively stable frontier orbital energies across mutants, underscoring the limited discriminatory power of HOMO–LUMO metrics in this context. Many orbital energies were much lower than −1.5, suggesting higher electronegativity within these atomic regions, though it is apparent that, across samples, the electronic stability across atoms is consistent among all mutants.

**FIGURE 10:**
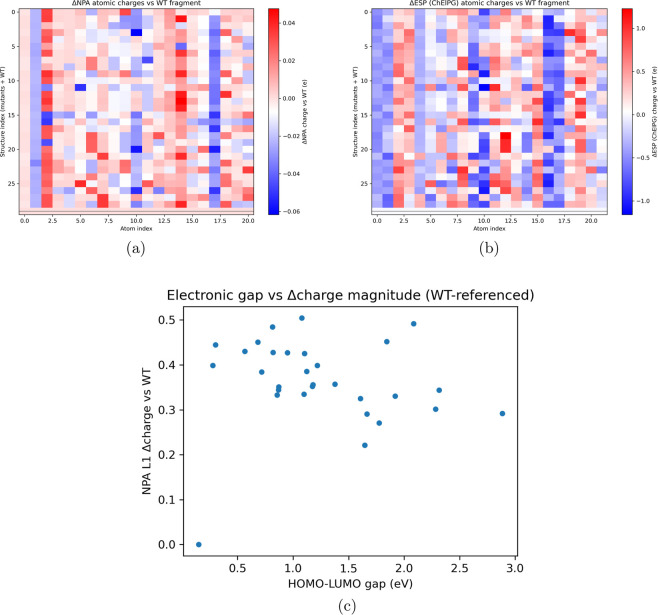
Quantum-chemical perturbation of TP53 mutants relative to the wild-type (WT) fragment. **(a)** and **(b)** show WT-referenced changes in NPA and ESP atomic charges, respectively, highlighting residues and environments with strong electronic redistribution. **(c)** links frontier-orbital perturbation (HOMO–LUMO gap) to the total magnitude of charge reorganization, summarizing how severely each mutant distorts the local electronic structure relative to WT. **(a)** ANPA atomic charges vs WT fragment. Each row corresponds to a TP53 mutant (in- cluding WT), each column to an atom index in the QM fragment; colors indicate the change in NPA charge relative to WT. **(b)** AESP (ChEIPG) atomic charges vs WT fragment. Each row corresponds to a TP53 mu- tant; colors indicate the change in ESP-derived partial charge relative to WT. **(c)** Relationship between HOMO-LUMO gap (eV) and the magnitude of the WT-referenced charge perturbation (L1 norm of Acharge vector). Each point is a TP53 mutant; larger values indicate stronger electronic disruption relative to WT.

In order to compare the unstable mutants to the wild type, differences between the quantum electronic properties of each mutant and the wild type were graphed for NPA, ESP, and HOMO/LUMO analyses in Figure 10. Across each atom in the mutant structures, there is consistently higher electronic sparsity compared to the wild type, with the exception of indices 10 and 20, which likely correspond to hydrogenous regions. The ESP values for the mutants were lower than those of the wild type across most variants. This suggests that electronic sparsity and reduced electrostatic potential may contribute to destabilization of DNA-binding or tetramerization regions by decreasing electronegativity in these regions. The orbital energies were much higher in the mutants than in the wild type, indicating lower electronegativity across the mutants. While these conclusions are limited by low-confidence ColabFold generations, they remain consistent with prior literature on mechanisms of TP53 affinity and structural destabilization.

The study presents a successful synthesis of mutant PDB forms, with electronic patterns characterized by lower electronegativity and sparse charge distribution. Likewise, there is lower electrostatic potential in these mutant types, reflecting low sample variability.

## Discussion

Population-level mutant analysis is possible using mass analysis of WXS sequencing. Transfer of these informatics into FASTA format and sequential ColabFold generation was successful, though confidence was unremarkable. Electrostatic potential was consistently lower in the mutant cohort due to the low sample size, and the electronic distribution was consistently more sparse, likely due to heavy residues and mutations with aromatic groups. Nevertheless, these mutations consistently exhibited lower electronegativity, which is compatible with previously reported destabilization mechanisms of DNA-binding domains and tetramerization regions. Orbital analyses demonstrated higher electronic stability within the mutants, and additional analyses can be performed to produce Raman spectra, chemical shifts, and nuclear magnetic resonance data across these fragments. These analyses can be used to perform cohort-level quantum characterization of gene mutations crucial in oncogenesis, more specifically, oncogenic processes.

Mass analysis of cancer cohort data can provide general mechanistic observations and characteristics regarding how mutations result in protein dysfunction. We generated structures for 28 mutants within the BRCA cohort using a developed pipeline and performed large-scale quantum chemical analysis to understand general trends in mutative behavior in TP53. For future studies, additional features can be developed specifically for high-confidence mutation prediction, in conjunction with physicochemical analysis.

## Limitations

This study is limited by the low-confidence generation of ColabFold structures and does not use all five biological replicates. While the Q-Chem analyses are complete, the limited sample size constrains statistical generalization, positioning this study as a feasibility analysis. The pipeline is also limited by the use of ColabFold-generated wild-type structures. MSA was not able to generate high-confidence evolutionarily similar proteins for prediction of high-confidence regions, such that in-depth analysis of mutations in other domains is limited. The limited sample size of the BRCA cohort means that mutation analyses are valid primarily with respect to replicating previous observations reported in prior studies.

## Data Availability

The datasets presented in this study can be found in online repositories. The names of the repository/repositories and accession number(s) can be found in the article/supplementary material. All code is available on GitHub: https://github.com/Alejandro21236/GenBlosum-WXSQCHEM.
